# Effects of Ultrasound Treatment on Emulsifying Properties of Pea Protein Isolates Obtained from Four Different Pea Flour Varieties

**DOI:** 10.3390/foods14091634

**Published:** 2025-05-06

**Authors:** Fatma Dadi, Ahmed Taha, Lucas Sales Queiroz, Naaman Francisco Nogueira Silva, Ipek Altay, Yuri Kominami, Rodolphe Marie, Aberham Hailu Feyissa, Jens J. Sloth, Heidi Olander Petersen, Mauro Grandi, Giorgia Spigno, Federico Casanova

**Affiliations:** 1 Department for Sustainable Food Process (DiSTAS), Università Cattolica del Sacro Cuore, Via Emilia Parmense 84, 29122 Piacenza, Italy; fatma.dadi@unicatt.it (F.D.); giorgia.spigno@unicatt.it (G.S.); 2 Department of Food Sciences, Faculty of Agriculture (Saba Basha), Alexandria University, Alexandria 21531, Egypt; ahmed-taha@alexu.edu.eg; 3 Research Group for Food Production Engineering, National Food Institute, Technical University of Denmark, Søltofts Plads, 2800 Kongens Lyngby, Denmark; lusaqu@food.dtu.dk (L.S.Q.); ipeal@food.dtu.dk (I.A.); abhfe@food.dtu.dk (A.H.F.); hope@food.dtu.dk (H.O.P.); 4 Centro de Ciências da Natureza, Universidade Federal de Sao Carlos (UFSCar), Buri 18245-000, Sao Paulo, Brazil; naaman.nogueira@ufscar.br; 5 Graduate School of Agricultural and Life Sciences, The University of Tokyo, Bunkyo-ku, Tokyo 1138657, Japan; akomi@g.ecc.u-tokyo.ac.jp; 6 Department of Health Technology, Technical University of Denmark, Ørstedsplads, 2800 Kongens Lyngby, Denmark; rcwm@dtu.dk; 7 Research Group for Analytical Food Chemistry, National Food Institute, Henrik Dams Alle B201, 2800 KGS Lyngby, Denmark; jjsl@food.dtu.dk; 8 HiWeiss S.r.l., Via A. Volta 13/A, NOI Techpark, 39100 Bozen, Italy; alfalfa.amministrazione@gmail.com

**Keywords:** pea protein, ultrasound, emulsions, physicochemical properties, FTIR

## Abstract

This study investigated the extraction and colloidal properties of pea protein isolates (PPIs) from four pea cultivars produced in a southern region in Italy. The control PPIs (C-PPIs) were obtained via an alkaline extraction and isoelectric precipitation method and then subjected to ultrasound treatment (US) to yield US-PPIs. The effects of cultivars and sonication on the physicochemical characteristics and emulsifying property of the PPIs were studied. Fourier-transform infrared (FTIR) spectroscopy, colorimetric measurement, dynamic light scattering (DLS), and confocal laser scanning microscopy (CLSM) were applied to characterize the samples. DLS results showed that C-PPIs displayed smaller particle sizes (8.86–15.9 µm) in comparison to US-PPIs (15.8–66.5 µm). DSC data showed that US-PPIs had improved thermal stability compared to control PPIs. FTIR analysis detected differences in the protein secondary structure of the various cultivars and between the native and US-PPIs. Emulsion stability studies indicated that emulsions stabilized with C-PPIs exhibited lower droplet sizes, implying improved stability in comparison to emulsions stabilized with US-PPIs. In conclusion, PPIs can be successfully extracted from different cultivars and applied as a natural emulsifier.

## 1. Introduction

Plant proteins have emerged as an alternative source to animal proteins due to their lower environmental footprint, and health benefits [1]. Among plant proteins sources, pea (*Pisum sativum*) is considered a novel protein source in several food applications, for example, as emulsifiers and foaming agents. Pea includes four protein groups, including albumin, globulin, glutelin, and prolamin, in which albumin and globulin are the major storage proteins in seeds [2]. Besides their nutritional value, pea protein isolates (PPIs) offer various functionalities due to their emulsifying and gelling properties [3]. The protein solubility plays an essential role on its emulsifying performance [4]. Several factors can impact the solubility of PPIs as a primarily globular protein, including (a) dissociation and association of subunits; (b) the existence of acidic, basic, and hydrophobic subunits on the protein surface; and (c) variations in molecular flexibility and molecular weight [5]. Thus, pea protein solubility is highly affected by the extraction and processing conditions, and consequently, PPI-stabilized emulsions will be sensitive to changes in the medium’s pH [6]. Thus, new protein extraction methods and processing technologies are recommended to improve the functional properties of these proteins.

Salt extraction dialysis, micellar precipitation, and alkaline extraction followed by isoelectric precipitation are the main methods applied for plant protein extraction. The alkaline extraction followed by isoelectric precipitation method is widely used for protein extraction due to its simplicity and relatively low cost, making it the most preferred approach currently [7]. However, it is worth noting that extreme alkaline conditions during extraction could lead to excessive denaturation of the extracted proteins and then changes in their functionality. For instance, Gao et al. [8] concluded that increasing pH can promote protein aggregation and reduce protein solubility and suggested that pH 9 was the optimum pH for PPI extraction.

Several processing techniques have been used to assist in the extraction of biomolecules from plants. Ultrasounds have been applied as a pre-extraction method to enhance the permeability of the cell wall, facilitating the extraction of proteins and phytochemicals [9]. Ultrasonic waves moving through a liquid create an acoustic cavitation phenomenon, where bubbles grow gradually and then collapse due to changes in temperature and pressure [10]. These phenomena contribute to the rupture of plant cells during protein extraction from seed flour [11]. Several authors found that ultrasounds were successfully utilized to assist the extraction of proteins from plant sources [12,13,14,15].

In this work, we investigated the influence of different pea cultivars on the protein recovery and chemical composition of PPIs obtained through an alkaline extraction followed by an isoelectric precipitation process. Furthermore, a post-extraction ultrasound treatment was applied to investigate the effect on functional properties of the previously obtained PPIs. The results of this work could assist researchers, food industries, and field crops companies in selecting the best cultivars and the most appropriate techniques for plant protein extraction and functionality improvement.

## 2. Materials and Methods

### 2.1. Material

Whole grains from four commercial pea varieties registered in France (V1—Ball Trap, registration number FDP04505-03; V2—Curling, registration number FDP20; V3—Escrime, registration number FDP10506; V4—Paddle, registration number FDP11514), cultivated in 2021 in Southern Italy (Foggia Province), were provided by Florimond Desprez Italia (Italy). V1, V2, and V3 are yellow peas, while V4 is a green pea.

**Figure 1:**
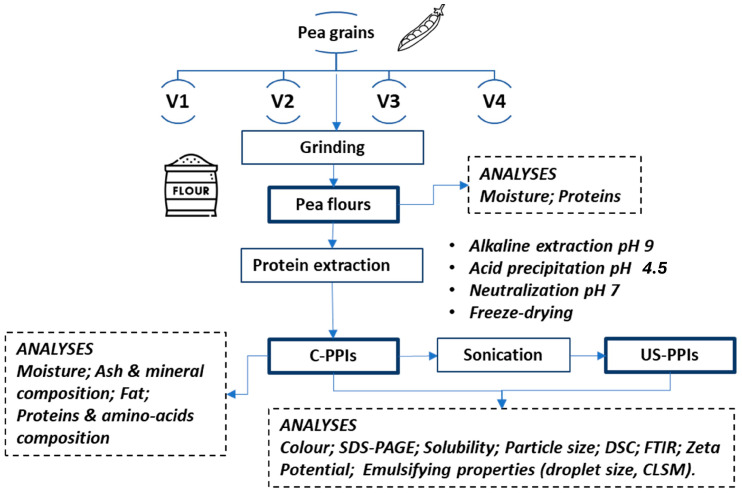
Schematic diagram represents the experimental plan of the study for protein extraction and sonication from different pea varieties.

The experimental plan is reported in Figure 1. Shortly, the pea varieties were characterized for moisture and protein content. Freeze-dried control PPIs (C-PPIs) were obtained through an extraction protocol based on alkaline extraction and acid precipitation, followed by neutralization and freeze drying. The C-PPIs were analyzed for total mass, content of moisture and fat, total proteins content and amino-acid composition, ash content, and mineral composition. The C-PPI were subjected to a sonication treatment to obtain the US-PPIs. Both control and sonicated samples were analyzed for color, SDS-PAGE, solubility, particle size, differential scanning calorimetry (DSC), Fourier transform infrared spectroscopy (FTIR), and ζ-potential. Emulsifying properties were investigated by measuring the droplet size, and morphological images were acquired by using confocal laser scanning microscopy (CLSM).

### 2.2. Extraction Protocol

The extraction of pea protein from *Pisum sativum* grains was performed according to [8] and the HiWeiss patent (EP 4 076 000 A1, A process for the preparation of undenatured vegetable proteic isolates) (Figure 1). Briefly, the process began with the milling of pea grains with Bühler Laboratory Disk Mill DLFU to yield a powder with a particle diameter of 0.5 mm. The pea flour was then mixed with water, and the pH of the resulting solution was consistently measured and adjusted to 9.0, indicating the initiation of the alkaline extraction phase. The mixture was heated to 50 °C and maintained at this temperature for 30 min under stirring. Following the heating, the mixture underwent centrifugation at 2500× *g* for 15 min. This step separated the liquid phase, containing the solubilized proteins, from the solid residue. The protein-rich liquid was then acidified to reach a pH of 4.5 (p*I*) to obtain protein precipitate. Precipitate proteins were separated by centrifugation at 2500× *g* for 25 min. The precipitated proteins were solubilized (1:10 *w*/*w*) in distilled water, neutralized to pH 7, and freeze-dried, yielding the C-PPIs, which were stored at 5 °C until further use and analyses.

### 2.3. Ultrasound Treatment

A 1% *w/w* suspension of pea protein (pH 7.0) was prepared in 50 mL of distilled water for the ultrasound treatment. The treatment was accomplished using a probe-type ultrasonicator (Branson Digital Sonifier SFX 550, EMERSON, St. Louis, MO, USA) with the following settings: 20 kHz frequency, 90% amplitude, 550 W power, and 20 min of pulsed mode with 5 s “ON” and 5 s “OFF” cycles. Throughout the treatment process, the entire solution was maintained at 35 °C in a water bath. After US treatment, the suspension was then centrifuged at 2500× *g* for 25 min, and the precipitated proteins were freeze-dried and stored at 5 °C until further analysis.

### 2.4. Analytical Methods

#### 2.4.1. Protein, Moisture, Ash, and Fat Content

The total crude protein content was determined using the Dumas method (Elementar, rapid MAX N EXCEED, Langenselbold, Germany) applying a nitrogen conversion of 6.25. Moisture and ash content were determined with AOAC standard methods 950.46 and 938.08, respectively (AOAC, 1990). The analyses were performed in duplicate.

#### 2.4.2. Amino Acids Composition

Acid hydrolysis (6 M HCl) in an oven for 18 h at 110 °C using 10 mg sample per mL of HCl was applied for amino acid profile measurement. Samples were then cooled at room temperature (25 °C) and used both without dilution and after a 3-fold dilution with 6 M HCl to quantify, respectively, low- and high-abundant amino acids. During acid hydrolysis, cysteine and tryptophan are destroyed, and therefore, they are not reported in the results. Next, 100 μL was diluted with 1.5 mL 1 M NaCO_3_ and filtered in a 0.2 μm syringe filter (Q-max PTFE, Ø13 mm, Frisenette ApS, Knebel, Denmark) before derivatization using the EZ: Faast™ Amino Acid Analysis kit from Phenomenex (Torrance, CA, USA). Then, 50 μL of samples were analyzed by LC-(APCI)-MS (Agilent 1100, Agilent Technology, Santa Clara, CA, USA) [16]. The analyses were realized in triplicate.

#### 2.4.3. Mineral Composition

The content of the elements was determined, following the method of Ozaktan et al. [17], with minor modifications, by either inductively coupled plasma mass spectrometry (ICAP TQ ICP-MS) for the microelements (Al, Cr, Fe, Mn, Ni, Cu, Zn, Sr, and Ba) or inductively coupled plasma optical emission spectrometry (Agilent 5800 ICP-OES) for the macroelements (Ca, K, Na, P, and S) following microwave-assisted digestion (Multiwave 7000, Anton Paar, Graz, Austria) using concentrated nitric acid (SPS Science, Paris, France). The sample digests were diluted with ultrapure water (milli-q) prior to analysis. All samples were analyzed in duplicate. Quantification was performed by external calibration with internal standardization using rhodium (Rh) and bismuth (Bi) as internal standards (ICP-MS) or yttrium (Y) (ICP-OES). All standards were prepared from certified stock solutions (SPS Science). Certified reference materials (NIST 1572 Citrus leaves and DORM-5 fish protein) were used for quality assurance of the analytical results.

#### 2.4.4. Color Measurement

The color of both C-PPIs and US-PPIs was analyzed using the colorimeter Minolta Chroma Meter CR-300 to obtain the CIELAB color space coordinates L* (brightness, where 100 is the perfect white, and 0 is black), a* (negative values representing green and positive values red), and b* (negative values representing blue and positive values yellow) [18]. The analyses were carried out in duplicate.

#### 2.4.5. SDS-PAGE

Sodium dodecyl sulphate poly acrylamide gel electrophoresis (SDS PAGE) was performed to determine the protein profile of PPIs using Laemmli’s method [19]. The method was applied in a Mighty Small (Hoefer) slab cell using 12% acrylamide (C = 2.6% (*w/w*)) slab gels (1.5 mm thick). Volumes of 2 mL 1% sodium dodecyl sulfate (SDS), 100 mM dithiothreitol (DTT), and 60 mM Tris–HCl (pH 8.3) were used for extraction of the dry sample (50 mg). The samples were prepared by shaking at room temperature for 1 h, followed by homogenization with Polytron PT 1200, Kinematica for 30 s, boiling for 2 min, and cooling at room temperature for 30 min. Then, the supernatants were diluted with buffer 125 mM Tris–HCl at 6.8 pH, 2.4% SDS, 50 mM DTT, 10% *v/v* glycerol, 0.5 mM EDTA, and bromophenol blue. Samples were loaded into the samples well, and the proteins were separated by applying 100 V constantly for 15 min, followed by 150 V for 1 h. The gels were then stained with Coomassie Brilliant Blue by molecular weight marker (Mark 12TM from Novex, Thermo Fisher Scientific, Waltham, MA, USA). Washing and revelation was performed by gel soaking into acetic acid solution 10% (*w/w*).

#### 2.4.6. Solubility

The solubility assay was carried out in accordance with Lv et al. [12] with a few minor adjustments. Suspensions of PPIs (1% in distilled water, based on protein content) at pH 7 were prepared and centrifuged for 15 min at 10,000× *g* at 4 °C. Using the Dumas, the nitrogen content of the protein dispersion prior to centrifugation and of the final supernatant were determined [20]. The following equation was used to determine the solubility:

(1) Solubility(%)=(Ns/Nt) × 100

where Ns is the amount of protein in the supernatant, and Nt is the total amount of protein prior to centrifugation. The analyses were performed in duplicate.

#### 2.4.7. Particle Size Analysis

The particle size distribution was evaluated on PPIs suspensions (1% *w/w* in distilled water, based on protein content) using a Mastersizer Malvern 2000 particle size analyzer [21]. All measurements were performed in triplicate at 25 °C.

#### 2.4.8. Fourier Transform Infrared Spectroscopy (FTIR)

The secondary structure of both C-PPIs and US-PPIs was analyzed using a PerkinElmer Spectrum 100 FT-IR spectrometer (Waltham, MA, USA). Each sample’s FTIR spectra (analyses performed in duplicate) were recorded with a resolution of 1 cm^−1^. The spectra were analyzed using the Origin 2021 software (OriginLab, Northampton, MA, USA).

#### 2.4.9. Zeta-Potential

ζ-Potential of C-PPIs and US-PPIs was assessed on 1% *w/w* (based on proteins content) suspensions in distilled water using a Zetasizer Nano-ZS (Malvern Instruments, Worcestershire, UK) [22]. Briefly, the samples were diluted 10 times in distilled water and placed in capillary cells (Malvern Instruments, Worcestershire, UK). After allowing the samples to equilibrate for 5 min, the analysis was recorded at 22 °C. Analyses were realized in duplicate.

### 2.5. Emulsifying Properties

Emulsions were prepared using PPIs (0.5% *w/v* based on protein content) and commercial sunflower oil (5% *v/v*, Vita d’Or^®^ purchased in local shops) in distilled water. Initially, pea protein powder was dispersed in distilled water under stirring at room temperature for one hour for the hydration process and then refrigerated at 4 °C overnight. The mixture was then stirred and oil gradually added drop by drop under continuous mixing using an Ultraturrax at 16.000 rpm for 3 min.

#### 2.5.1. Emulsion’s Droplet Size and ζ-Potential

The droplet size distributions of the PPI emulsions were analyzed using a Mastersizer Malvern 2000 particle size analyzer [23]. All measurements were conducted at 25 °C. Analyses were carried out in duplicate.

#### 2.5.2. Confocal Laser Scanning Microscopy (CLSM)

Fluorescence Confocal Laser Scanning Microscopy (CLSM) was used to observe the microstructure of the emulsions prepared with C-PPIs or US-PPIs. Nile Red and FCF Fast Green dyes (both from Sigma-Aldrich Denmark A/S, Søborg, Denmark) were added at a concentration of 0.01% and 0.001%, respectively, to label the fat droplets and proteins [24]. The imaging system used in this study was a confocal spinning disc microscope, which consisted of an inverted microscope (Nikon Ti2), a laser source emitting light at wavelengths of 405/488/561/640 nm, a confocal spinning disc module (Yokogawa CSU-W1) with 50 μm pinholes, two single-band emission filters (600/50 nm and 700/75 nm), and a sCMOS camera (Photometrics Prime95B). Nile Red (the fat droplets) was imaged using a 561 nm excitation and emission at 600/50 nm. FCF Fast Green (proteins) was imaged at a 640 nm excitation and an emission at 700/75 nm. Analyses were carried out in duplicate.

### 2.6. Statistical Analysis

Extractions and sonication were carried out in duplicate. Each analysis was carried out either in duplicate or triplicate, as previously specified. The results were reported as the average value ± the standard deviation. The differences between means were assessed using one-way analysis of variance (ANOVA) and Tukey’s paired comparison test. A significance level of 0.05 was used for all analyses.

## 3. Results and Discussion

### 3.1. Effect of Variety on Protein Recovery, Proximate Analysis, and Mineral Composition

Protein recovery indicates the efficiency of the extraction process. Table 1 shows the protein recovery and the protein content in PPI. The results indicated that cultivars 1 and 4 (52.8 and 51.2%, respectively) had significantly higher extraction yield compared to those of cultivars 2 and 3 (42.6 and 46.1%, respectively). Moreover, cultivar 1 had a protein content of 88.4%, which was lower but not significantly compared to other cultivars (around 91% protein). For the fat content, cultivars 1 and 4 had higher fat content than other cultivars. The mineral composition of the extracted PPI is shown in Table 2. It was found that phosphorus and sulfur were the most abundant macroelements detected in PPI from different cultivars. In contrast, calcium and sodium were the least abundant macroelements with concentrations below 1.8 g/kg. For the microelements content of extracted PPI, iron and zinc were found to be present at higher concentrations than other micronutrients. Furthermore, cultivar V3 had the highest content of Fe and Zn. The varying mineral concentrations in pea flour from distinct cultivars may result from the mineral composition of the soil in which the pea plants were cultivated. Moreover, the fertilization protocol used could have an impact on the final mineral composition of the pea flour [25].

### 3.2. Effect of Ultrasound Treatment and Variety on Protein Quality and Functionality

#### 3.2.1. Protein Molecular Weight Distribution and Amino Acid Profile

**Figure 2:**
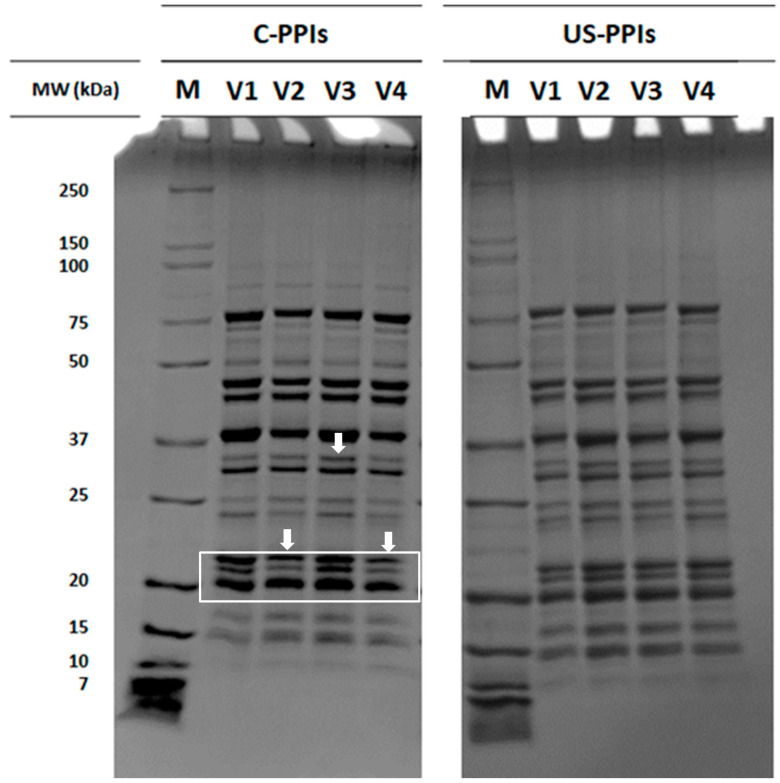
SDS-PAGE of C-PPIs and US-PPIs.

The protein patterns of the extracted PPI before and after ultrasound treatment are shown in Figure 2. The protein profile’ s pattern of C-PPIs and US-PPIs samples showed a wide range of protein molecular weights, spanning from 7 to 250 kDa. Bands with a molecular weight below 15 kDa are classified as part of the albumin fraction. The presence of bands at 18, 35, and 50 kDa suggests the association of vicilin trimers, whereas a band around 75 kDa indicates the presence of convicilin [26]. Similar patterns were reported with PPI [27], confirming the high efficiency of protein extraction. In C-PPIs samples, some differences among cultivars were observed in the band intensity between 20 and 37 kDa. No major differences were detected in the protein SDS-PAGE profile between C-PPIs and US-PPIs samples. Similar findings were stated by [28], who found ultrasound was not able to induce major changes in the SDS-PAGE patterns of black bean protein. The amino acid profile of different pea cultivars is presented in Table 3. The results showed PPI had a low methionine (MET) content but a high content of glutamate (GLU), aspartate (ASP), and arginine (ARG). Similar findings were reported by [29], who found that pea protein-enriched flour is rich in GLU and ASP amino acids. Different cultivars had similar amounts of each amino acid. The amino acid profile of different pea cultivars is presented in Table 3. In general, pea proteins are particularly rich in essential amino acids such as lysine, arginine, and branched-chain amino acids (BCAAs) like leucine, isoleucine, and valine, which play critical roles in muscle protein synthesis and metabolic health [30]. However, pea proteins are reported to present limited amounts of sulfur-containing amino acids (methionine and cysteine), which can affect their protein quality unless combined with complementary protein sources. The results shown in Table 3 are in line with the literature, as the four varieties of pea presented low sulfur-containing amino acid content and high contents of the other essential amino acids. Regarding the non-essential amino acids, similar findings were reported by [29], who found that pea protein-enriched flour is rich in glutamic and aspartic acids. With respect to the essential amino acid score (EAA), pea proteins are comparable with soy proteins in terms of nutritional value, presenting values close to 1.0 or slightly above [31].

#### 3.2.2. Colorimetry

Table 4 shows the colorimetry analysis for C-PPIs and US-PPIs powder. The b* value ranges from negative values for blueness to positive values for yellowness. The a* value represents the level of redness with positive values and the amount of greenness with negative values. On the other hand, the L* value represents the degree of brightness, ranging from 0 (completely dark) to 100 (full brightness) [32]. In C-PPIs, V4 had a darker color with lower L* value compared to other cultivars. The results showed brighter color for the ultrasound-treated samples with greater L* values than C-PPIs samples. Moreover, a* values indicated a shift towards green color following ultrasound treatment in all cultivars except V4. The ST-V4 sample also had a lower b* value compared to other cultivars, indicating a minor shift towards blue color. Furthermore, ultrasound-treated samples had lower b* values compared to standard PPI samples. Ultrasound can affect proteins’ colors positively or negatively. It may accelerate the release of pigment from protein suspensions and degrade pigment-containing sites which absorb light [33]. Ultrasound-treated samples were significantly lighter (Table 4) than the standard ones, and it was also visually noticeable that the suspensions became whiter and less yellowish after US treatment. It is well known that US treatment can induce the breakdown of plant structures, which was probably related to the increased light scattering of US-treated samples.

#### 3.2.3. Particle Size

**Figure 3:**
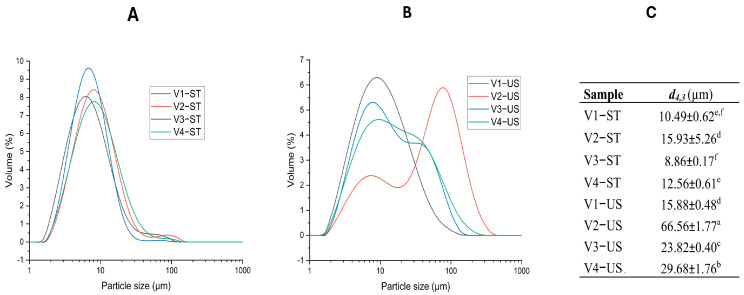
Particle size distribution (PSD) of C-PPIs (**A**), US-PPIs (**B**), and volume surface mean diameter (*d_4,3_*) of P C-PPIs (**C**). Values in each column with different letters are statistically significant (*p* < 0.05).

The volume mean diameter (*d*_4,3_) and particle size distribution (PSD) of C-PPIs and US-PPIs are shown in Figure 3C, respectively. PPIs from cultivars V2 and V4 had bigger particle sizes (*d*_4,3_) than V1 and V3 cultivars (Figure 3C). Additionally, ultrasonic-treated proteins had significantly bigger particle sizes than C-PPIs proteins. V2-US-PPI showed the biggest particle size (66.56 ± 1.77 µm). The PSD results supported these findings where the V2-US sample had a bimodal size distribution, indicating potential protein aggregation. This might occur because some protein molecules in variant V2 might form complexes with other compounds (i.e., polysaccharides) in pea flour, thus increasing their particle sizes; this is matched with the high protein content’s slightly lower percentage of protein recovery (Table 1) due to potential binding of proteins to other macromolecules in the V2 variant. However, further studies are required to confirm this hypothesis. Some studies reported that sonication could reduce the particle size of proteins [34,35]. The reduction in the particle size after sonication could probably occur because acoustic cavitation could disassemble the compact protein structure and disrupt the intermolecular interactions. This could dissociate protein aggregates into monomers, reducing the particle size [34]. In the current research, the observed significant increase in particle size after ultrasound treatment could be attributed to the disruption followed by aggregation of the protein microstructure due to acoustic cavitation. This disruption results in the swelling of protein particles when dispersed in water or the formation of protein self-assemblies because of hydrophobic interactions among the unfolded regions [36].

#### 3.2.4. Secondary Structure

**Figure 4:**
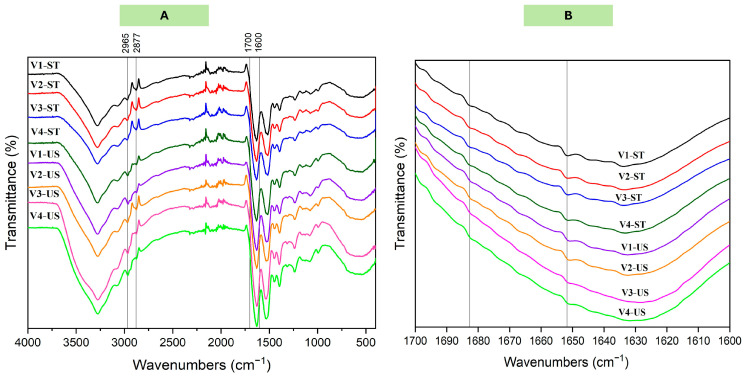
(**A**) The FTIR spectra of C-PPIs and US-PPIs samples extracted from different cultivars (V1–V4). (**B**) FTIR spectra in the amide I (1600–1700 cm^−1^) vibrational range.

FTIR spectroscopy is an effective method for obtaining biochemical fingerprints and identifying structural alterations in proteins. The FTIR can be used to analyze functional groups and secondary protein structures to determine the molecular structure and composition [37]. The amides I, II, and III play a crucial role in assessing the level of molecular organization in proteins and contribute to the development of protein structures. The alterations observed in the amide I region (1600–1700 cm^−1^) signify modifications in the stretching of the C=O bond. The amide II region (1500–1600 cm^−1^) corresponds to the bending of N-H bonds and the stretching of C-H bonds. Both amide I and amide II regions are responsive to alterations in the secondary structures of proteins [38]. Some differences were observed in the peaks’ intensities of different PPI cultivars before and after sonication at 2965 and 2877 cm^−1^ (Figure 4A), indicating minor changes in the symmetric and asymmetric CH_2_ stretching modes [39]. Moreover, the peaks of different cultivars in the amide I region exhibited changes in the intensities after sonication, indicating changes in the secondary structures of the PPI following ultrasound treatment [40]. The Peakfit software (version 4.12, Seasolve Software Inc., Palo Alto, CA, USA) was utilized to detect concealed peaks within the amide I (1600–1700 cm^−1^) region (Figure 4B), with the aim of acquiring insights into the alterations in protein secondary structure. Table 5 represents the secondary structure composition of C-PPIs pea proteins from different cultivars. Cultivar V4 showed the highest β-sheet content in both C-PPIs and US-PPIs. Moreover, the random coil content of PPI from different cultivars increased after US treatment. In cultivars V1–V3, the β-sheet content decreased significantly after US treatment. Similar findings were observed with soy protein isolate, in which US treatment increased the random coil and reduced the β-sheet contents [41]. The disruption of the hydrogen bond in the protein led to a decrease in the presence of organized structures inside the protein molecule, resulting in the unfolding of the PPI and a loosening of the molecular structure. The acoustic cavitation impact of sonication alters the secondary structure of proteins by disrupting the intermolecular hydrogen bonds and enhancing protein flexibility [42].

### 3.3. Emulsifying Properties

#### 3.3.1. Microstructure of Protein Particles and Emulsions

**Figure 5:**
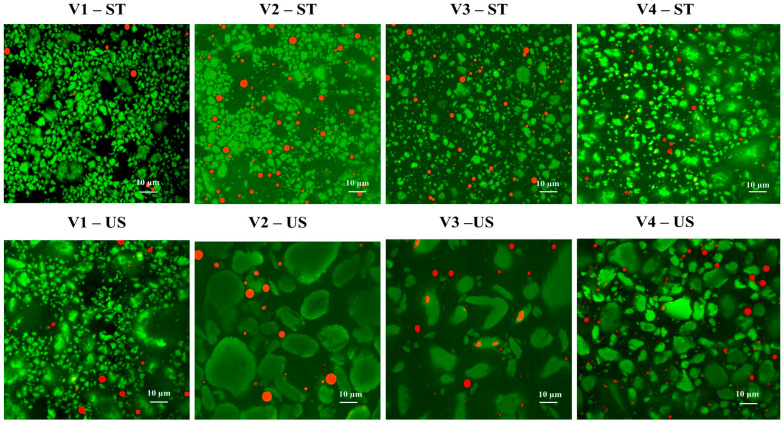
Confocal laser scanning microscopy (CLSM) of emulsions stabilized by C-PPIs and US-PPIs extracted from different cultivars (V1–V4). The Fast Green signal (green) and Nile Red signal (red spots) represent the protein and fat, respectively.

CLSM is commonly employed for the examination of microstructure and interfacial characteristics of emulsions [43]. The CLSM micrographs presented in Figure 5 show that US-treated PPI of the cultivars V2–V4 had bigger protein aggregates compared to the C-PPIs. These findings are in line with the particle size results (*d*_4,3_) of PPI (Figure 3), where most of the US-PPIs exhibited significantly larger particles than C-PPIs. The V2-US sample had the largest particle size (66.56 ± 1.77 µm) (Figure 3C), which corresponded to the large protein aggregates observed in the CLSM image (V2-US, Figure 5). Moreover, the CLSM images show that the oil droplets of emulsions stabilized using US-treated PPI exhibited slightly bigger oil droplets.

#### 3.3.2. Droplet Size and Zeta-Potential

The oil droplet size of an emulsion is a crucial characteristic that affects its stability and physicochemical characteristics [44]. As presented in Table 6, the emulsion samples stabilized using native PPI had smaller oil droplets compared to those stabilized using US-treated PPI. Moreover, the emulsion stabilized using the V3-ST protein sample had the smallest oil droplet size (10.66 ± 0.70 µm). This could be attributed to the small protein particles of the V3-ST protein, as shown in Figure 3. The small particle size of protein before emulsification led to a decrease in droplet size of emulsions. Smaller protein particles could move faster towards the interface between water and oil. Additionally, the smaller protein particles enhanced the absorption rate of PPI on the surface of oil droplets, thereby preventing coalescence, flocculation, and gravitational separation [45]. The ζ-potential is a measure of the level of electrostatic repulsion between neighboring particles in an emulsion system. It is an important indicator of the stability of the emulsion, as a greater absolute zeta-potential value of an emulsion means a higher level of stability [46]. The ζ-potential values (Table 6) revealed that emulsions stabilized by native PPI extracted from variants V1–V3 had the higher absolute ζ-potential values compared to those stabilized US-treated PPI from cultivars (V2–V4). This indicates that native PPIs could produce more stable emulsions. Some researchers have reported that ultrasound treatment could reduce the particle size of proteins, and thus, US-induced protein emulsions had small oil droplets and high stability [45,47]. However, it was concluded that ultrasound treatment failed to improve the emulsifying properties of sodium caseinate and whey protein isolate [48]. In the current study, the high ultrasound amplitude (90%) and the long processing time (20 min) might have negatively affected the emulsifying performance of PPI. The alternations of secondary structure following ultrasound treatment (Table 5) may have led to the hiding of hydrophobic groups in protein molecules, thus lowering the emulsifying capabilities of the proteins. Moreover, the large particle size of ultrasound-treated proteins (Figure 3C) slowed the movement of protein molecules to the oil/water interface during emulsification, thus increasing the size of oil droplets of emulsions [10].

## 4. Conclusions

In this work, pea protein isolates were extracted using alkaline extraction–isoelectric precipitation method from four different commercial pea cultivars (V1–V4) cultivated in a southern region in Italy. The extracted PPIs were treated with ultrasound to investigate the potential effect on protein functionalities. Different cultivars and ultrasound treatment had different effects on the physicochemical and emulsifying properties of PPIs. The colorimetry and dynamic light scattering results showed that C-PPIs had darker color and smaller particle sizes compared to ultrasound-treated samples. FTIR data confirmed some differences in the secondary structure among different cultivars and between C-PPIs and US-PPIs. Following US treatment, PPI showed higher random coil content, and cultivars V1–V3 showed a significant decrease in β-sheet structures. In addition, the emulsions stabilized using C-PPIs had smaller droplets, indicating better stabilizing efficacy compared to US-treated PPIs. For future investigations, it could be recommended to investigate the impact of applying ultrasound technology at different parameters (i.e., intensity and duration) on the physicochemical and emulsifying properties of pea proteins. Moreover, it is suggested to compare the impact of ultrasound treatment of pea protein structure and functionality before and after protein extraction.

## Figures and Tables

**Table 1:** Composition of pea protein isolates (PPI) and of initial pea flour and protein recovery in the PPI. Values are reported as means ± standard deviation (s.d.). Values in each column with different letters were statistically significant (*p* < 0.05).

Variety	PPI	Flour				
	Moisture (% *w*/*w*)	Ash (% *w*/*w*)	Fat (% *w*/*w*)	Proteins (% *w*/*w*)	Proteins (% *w*/*w*)	Proteins Recovery from Flour (%)
**V1**	5.77 ± 0.03 ^d^	2.83 ± 0.04 ^a^	1.81 ± 0.01 ^a^	88.39 ± 1.7 ^a^	23.90 ± 0.02 ^c^	52.85 ± 2.3 ^a^
**V2**	7.13 ± 0.02 ^a^	2.57 ± 0.13 ^b^	0.98 ± 0.00 ^c^	91.67 ± 0.9 ^a^	25.24 ± 0.14 ^a^	42.61 ± 2.4 ^c^
**V3**	6.50 ± 0.02 ^c^	2.27 ± 0.03 ^c^	1.55 ± 0.02 ^b^	91.91 ± 1.3 ^a^	23.94 ± 0.09 ^c^	46.10 ± 0.6 ^b^
**V4**	6.63 ± 0.02 ^b^	2.78 ± 0.04 ^a^	1.83 ± 0.02 ^a^	91.24 ± 1.1 ^a^	24.44 ± 0.24 ^b^	51.20 ± 2.1 ^a^

**Table 2:** Mineral composition of control pea protein isolates (PPI), with values reported as means ± standard deviation (s.d.).

	ELEMENT	V1	V2	V3	V4
MACROELEMENTS (g/kg)	Ca	1.86 ± 0.03	1.47 ± 0.01	1.21 ± 0.02	1.69 ± 0.00
	K	2.80 ± 0.04	2.57 ± 0.04	2.33 ± 0.04	2.78 ± 0.02
	Na	0.15 ± 0.01	0.13 ± 0.00	0.12 ± 0.00	0.13 ± 0.00
	P	8.50 ± 0.05	8.27 ± 0.06	6.97 ± 0.06	8.72 ± 0.05
	S	4.49 ± 0.07	5.05 ± 0.06	5.18 ± 0.09	4.81 ± 0.01
MICROELEMENTS (mg/kg)	Al	4.10 ± 0.16	4.32 ± 0.01	4.09 ± 0.01	4.17 ± 0.29
	Cr	0.13 ± 0.12	0.17 ± 0.03	0.19 ± 0.07	0.17 ± 0.02
	Fe	147 ± 0.08	194 ± 3.9	200 ± 0.07	161 ± 3.69
	Mn	6.29 ± 0.08	5.86 ± 0.07	5.29 ± 0.00	5.78 ± 0.01
	Ni	1.41 ± 0.01	0.97 ± 0.04	1.02 ± 0.11	0.70 ± 0.00
	Cu	12.0 ± 0.11	8.52 ± 0.04	10.4 ± 0.05	11.5 ± 0.04
	Zn	38.2 ± 0.48	36.9 ± 0.20	41.3 ± 0.14	39.8 ± 0.16
	Sr	10.6 ± 0.11	7.60 ± 0.03	6.11 ± 0.02	8.35 ± 0.07
	Ba	0.99 ± 0.00	0.85 ± 0.04	0.62 ± 0.02	0.78 ± 0.00

**Table 3:** Amino acid composition of pea protein isolates from varieties V1, V2, V3, and V4. The essential amino acid (EAA) requirements (req*) were obtained from the WHO (2007). DM stands for dry matter.

	V1				V2				V3				V4				EAA req*
EAA	mg/100 g (DM)	% of Total	mg/g Protein	EAA Score *	mg/100 g (DM)	% of Total	mg/g Protein	EAA Score *	mg/100 g (DM)	% of Total	mg/g Protein	EAA Score *	mg/100 g (DM)	% of Total	mg/g Protein	EAA Score *	mg/g Protein
HIS	22.1 ± 1.4	2.7	23.5	1.57	23.2 ± 1.0	3.0	23.5	1.57	22.7 ± 1.1	2.7	23.1	1.54	23.3 ± 0.5	2.7	23.9	1.59	15
ILE	39.1 ± 2.6	4.7	41.7	1.39	40.3 ± 2.4	5.1	40.8	1.36	42.1 ± 1.2	5.0	42.9	1.43	40.4 ± 1.5	4.7	41.4	1.38	30
LEU	73.7 ± 4.0	8.9	78.6	1.33	76.0 ± 3.2	9.7	77.0	1.31	76.3 ± 3.2	9.0	77.6	1.32	75.8 ± 1.4	8.9	77.6	1.31	59
LYS	66.5 ± 2.8	8.0	70.9	1.58	68.1 ± 6.2	8.7	69.0	1.53	71.8 ± 2.2	8.5	73.0	1.62	66.9 ± 2.7	7.9	68.5	1.52	45
MET + CYS	8.9	1.1	9.5	0.43	9.6	1.2	9.8	0.44	11.4	1.3	11.6	0.53	7.5	0.9	7.7	0.35	22
MET	3.0 ± 1.8	0.4	3.2	0.20	4.0 ± 2.8	0.5	4.0	0.25	4.9 ± 2.0	0.6	4.9	0.31	2.3 ± 1.9	0.3	2.3	0.14	16
CYS	6.0 ± 0.5	0.7	6.3	1.06	5.7 ± 0.8	0.7	5.7	0.96	6.5 ± 0.5	0.8	6.6	1.11	5.3 ± 0.5	0.6	5.4	0.90	6
PHE + TYR	67.5	8.2	72.0	1.89	68.6	8.7	69.5	1.83	55.8	6.6	72.4	1.91	68.9	8.1	70.5	1.86	38
PHE	38.2 ± 1.8	4.6	40.7	-	38.5 ± 2.3	4.9	39.0	-	39.8 ± 1.2	4.7	40.4	-	38.8 ± 0.9	4.6	39.7	-	-
TYR	29.3 ± 2.3	3.5	31.3	-	30.1 ± 2.4	3.8	30.5	-	16.0 ± 2.5	1.9	32.0	-	30.1 ± 1.2	3.5	30.8	-	-
THR	29.3 ± 1.9	3.5	31.2	1.36	30.5 ± 1.7	3.9	30.9	1.35	30.7 ± 0.9	3.6	31.2	1.36	30.2 ± 0.6	3.6	30.9	1.35	23
VAL	42.3 ± 3.1	5.1	45.1	1.16	44.2 ± 1.5	5.6	44.8	1.15	44.3 ± 1.5	5.2	45.1	1.16	44.3 ± 1.2	5.2	45.4	1.16	39
Sub-total	349.4	42.2	372.5		360.6	46.0	365.3		355.0	42.0	376.9		357.5	42.0	365.9		
**NON-ESSENTIAL AMINO ACIDS**																	
ALA	35.2 ± 1.6	4.2	37.5		36.3 ± 1.7	4.6	36.8		36.9 ± 1.2	4.4	37.5		36.4 ± 0.9	4.3	37.3		
ARG	76.7 ± 5.3	9.3	81.8		11.0 ± 2.2	1.4	80.5		78.3 ± 5.0	9.3	79.7		80.6 ± 1.0	9.5	82.5		
APS	97.9 ± 4.1	11.8	104.4		99.2 ± 3.0	12.6	100.5		99.6 ± 3.8	11.8	101.4		99.8 ± 2.8	11.7	102.1		
GLU	151.8 ± 6.3	18.3	161.9		156.6 ± 5.7	20.0	158.7		156.4 ± 6.6	18.5	159.1		156.8 ± 3.7	18.4	160.5		
GLY	33.8 ± 1.3	4.1	36.1		35.0 ± 1.6	4.5	35.4		35.3 ± 1.4	4.2	35.9		34.7 ± 0.9	4.1	35.5		
PRO	38.3 ± 2.2	4.6	40.8		39.2 ± 1.2	5.0	39.7		39.1 ± 1.9	4.6	39.8		39.1 ± 0.6	4.6	40.1		
SER	45.3 ± 1.8	5.5	48.3		46.3 ± 1.7	5.9	46.9		45.5 ± 2.6	5.4	46.3		46.0 ± 0.8	5.4	47.1		
Total	828.5				784.1				846.2				851.0				

(*) Reference: World Health Organization. Protein and Amino Acid Requirements in Human Nutrition: Report of a Joint WHO/FAO/UNU Expert Consultation. (2007).

**Table 4:** Colorimetry analysis of C-PPIs and US-PPIs powders. Values are reported as means ± standard deviation (s.d.). Values in each column with different letters are statistically significant (*p* < 0.05).

	VARIETY	L*	a*	b*
**C-PPIs**	V1	71.37 ± 0.00 ^b^	−0.67 ± 0.04 ^b^	36.42 ± 0.01 ^a^
	V2	70.86 ± 0.00 ^b^	0.35 ± 0.03 ^a^	33.83 ± 0.00 ^b^
	V3	69.05 ± 0.00 ^b^	0.24 ± 0.01 ^a^	31.96 ± 0.00 ^b^
	V4	64.31 ± 0.00 ^c^	−5.73 ± 0.01 ^d^	29.94 ± 0.01 ^b^
**US-PPIs**	V1	77.25 ± 0.00 ^a^	−2.18 ± 0.02 ^c^	29.25 ± 0.00 ^b^
	V2	78.81 ± 0.01 ^a^	−2.96 ± 0.04 ^c^	24.42 ± 0.02 ^c^
	V3	79.93 ± 0.00 ^a^	−2.78 ± 0.00 ^c^	22.80 ± 0.00 ^c^
	V4	78.22 ± 0.00 ^a^	−4.92 ± 0.01 ^d^	23.16 ± 0.02 ^c^

**Table 5:** The secondary structure of C-PPIs and US-PPIs from different cultivars (V1–V4). Values in each column with different letters are statistically significant (*p* < 0.05).

Samples	Secondary Structure Composition (%)				
	β-Sheet Aggregates	β-Sheet	Random Coil	α-Helix	β-Turn
V1-ST	12.7 ± 1.2 ^b^	34.5 ± 2.2 ^b^	8.9 ± 1.8 ^c^	21.4 ± 2.3 ^a^	20.9 ± 1.9 ^b,c^
V2-ST	10.5 ± 1.4 ^b^	39.4 ± 3.1 ^c^	7.8 ± 1.5 ^c^	19.5 ± 3.4 ^a^	19.1 ± 2.2 ^c^
V3-ST	14.7 ± 0.8 ^a^	34.4 ± 1.8 ^b^	3.8 ± 0.5 ^c^	21.2 ± 1.2 ^a^	25.1 ± 0.8 ^a^
V4-ST	9.3 ± 0.9 ^c^	42.7 ± 2.4 ^a^	8.3 ± 0.7 ^c^	19.5 ± 2.1 ^a^	19.3 ± 2.4 ^c^
V1-US	11.9 ± 1.5 ^b^	25.1 ± 1.7 ^d^	14.1 ± 1.1 ^a^	21.5 ± 1.6 ^a^	19.9 ± 1.3 ^c^
V2-US	11.2 ± 1.1 ^b^	23.8 ± 2.3 ^d^	15.0 ± 1.5 ^a^	21.0 ± 1.4 ^a^	18.7 ± 3.1 ^c^
V3-US	12.3 ± 1.9 ^b^	24.8 ± 1.3 ^d^	16.3 ± 1.7 ^a^	22.6 ± 1.6 ^a^	21.2 ± 2.6 ^b^
V4-US	9.2 ± 0.7 ^c^	35.2 ± 3.5 ^b^	11.0 ± 1.3 ^b^	21.2 ± 2.4 ^a^	18.7 ± 1.6 ^c^
**Band frequency (cm^−1^)**	**1688–1700**	**1610–1642 and 1681–1687**	**1630–1645**	**1650–1663**	**1663–1680**

**Table 6:** Droplet sizes values of emulsions stabilized using C-PPIs and US-PPIs. ζ-potential values of emulsions using C-PPIs and US-PPIs. Values in each column with different letters are statistically significant (*p* < 0.05).

Sample	*d*_4,3_ (µm)	ζ-Potential (mV)
V1-ST	14.78 ± 0.46 ^d^	−23.06 ± 1.33 ^c^
V2-ST	11.50 ± 0.20 ^f^	−22.18 ± 1.30 ^c^
V3-ST	10.66 ± 0.70 ^g^	−22.15 ± 2.09 ^c^
V4-ST	13.16 ± 0.46 ^e^	−21.30 ± 1.11 ^b^
V1-US	19.33 ± 1.23 ^b^	−21.77 ± 0.68 ^b^
V2-US	17.36 ± 0.39 ^c^	−19.71 ± 1.78 ^a^
V3-US	21.02 ± 0.26 ^a^	−19.20 ± 1.27 ^a^
V4-US	15.07 ± 0.48 ^d^	−19.77 ± 1.38 ^a^

## Data Availability

The original contributions presented in this study are included in the article. Further inquiries can be directed to the corresponding author.
